# Application and Performance of Artificial Intelligence Technology in Oral Cancer Diagnosis and Prediction of Prognosis: A Systematic Review

**DOI:** 10.3390/diagnostics11061004

**Published:** 2021-05-31

**Authors:** Sanjeev B. Khanagar, Sachin Naik, Abdulaziz Abdullah Al Kheraif, Satish Vishwanathaiah, Prabhadevi C. Maganur, Yaser Alhazmi, Shazia Mushtaq, Sachin C. Sarode, Gargi S. Sarode, Alessio Zanza, Luca Testarelli, Shankargouda Patil

**Affiliations:** 1 Preventive Dental Science Department, College of Dentistry, King Saud Bin Abdulaziz University for Health Sciences, Riyadh 11481, Saudi Arabia; sanjeev.khanagar76@gmail.com; 2 King Abdullah International Medical Research Center, Ministry of National Guard Health Affairs, Riyadh 11481, Saudi Arabia; 3 Dental Biomaterials Research Chair, Dental Health Department, College of Applied Medical Sciences, King Saud University, Riyadh 11433, Saudi Arabia; snaik@ksu.edu.sa (S.N.); aalkhuraif@ksu.edu.sa (A.A.A.K.); 4 Department of Preventive Dental Sciences, Division of Pedodontics, College of Dentistry, Jazan University, Jazan 45142, Saudi Arabia; drvsatish77@gmail.com (S.V.); prabhadevi.maganur@gmail.com (P.C.M.); 5 Department of Maxillofacial Surgery and Diagnostic Sciences, Division of Oral Pathology, College of Dentistry, Jazan University, Jazan 45142, Saudi Arabia; dr.y.alhazmi@gmail.com; 6 College of Applied Medical Sciences, Dental Health Department, King Saud University, Riyadh 12372, Saudi Arabia; smushtaqdr@gmail.com; 7 Department of Oral and Maxillofacial Pathology, Dr. D.Y. Patil Dental College and Hospital, Dr. D. Y. Patil Vidyapeeth, Pimpri, Pune 411018, India; drsachinsarode@gmail.com (S.C.S.); gargi14@gmail.com (G.S.S.); 8 Department of Maxillo and Oro-Facial Sciences, University of Rome La Sapienza, 00185 Rome, Italy; ale.zanza@gmail.com (A.Z.); luca.testarelli@uniroma1.it (L.T.)

**Keywords:** artificial intelligence, artificial neural networks, oral cancer diagnosis, machine learning, oral cancer prediction

## Abstract

Oral cancer (OC) is a deadly disease with a high mortality and complex etiology. Artificial intelligence (AI) is one of the outstanding innovations in technology used in dental science. This paper intends to report on the application and performance of AI in diagnosis and predicting the occurrence of OC. In this study, we carried out data search through an electronic search in several renowned databases, which mainly included PubMed, Google Scholar, Scopus, Embase, Cochrane, Web of Science, and the Saudi Digital Library for articles that were published between January 2000 to March 2021. We included 16 articles that met the eligibility criteria and were critically analyzed using QUADAS-2. AI can precisely analyze an enormous dataset of images (fluorescent, hyperspectral, cytology, CT images, etc.) to diagnose OC. AI can accurately predict the occurrence of OC, as compared to conventional methods, by analyzing predisposing factors like age, gender, tobacco habits, and bio-markers. The precision and accuracy of AI in diagnosis as well as predicting the occurrence are higher than the current, existing clinical strategies, as well as conventional statistics like cox regression analysis and logistic regression.

## 1. Introduction

Oral cancer (OC) is one of the most common lethal diseases and has been a major public health concern around the world. OC is a subdivision of head and neck cancers with 275,000 fresh cases per year worldwide. The survival rate of the early stage (Stage I) disease is around 80%, whereas for the late stage disease (Stage II and III), it is less than 20% [1,2].

Among OC, squamous cell carcinoma (OSCC) of the oral cavity is the most common type and comprises 90% of the disease [3]. Early diagnosis of OC is significant, however, most patients are diagnosed at a late stage of the disease, leading to a poor prognosis. The clinical appearance of OC is not a sufficient parameter for identifying the status, analysis, or dysplastic level, therefore, the treatment selection based on the clinical appearance of the disease is not sufficient. OC is associated with multiple factors, and the survival rate after treatment is also unpredictable [4,5].

Potentially malignant lesions like leukoplakia, erythroplakia, and oral submucous fibrosis are also prevalent among the risk population. Differentiating these lesions from the malignant lesions are also important. Risk factors like age, gender, and tobacco habits may affect the prognosis of OC [6].

Understanding the refinements of innovations like Artificial Intelligence (AI) could relieve potential clinical entanglements [7,8]. Application of AI in the oral malignant growths can improve the current challenges in the disease diagnosis, as well as in predicting the prognosis. AI, which mimics human cognitive functions, is a forward leap in innovation, and has enamored the minds of scientists over the globe [9]. Its use in dentistry has begun recently, which has led to extraordinary accomplishments. History goes back to as early as 400 BC; Plato visualized an essential model of brain function. AI system is a framework that takes u information, discovers designs, uses data to train itself, and yields results [9,10,11].

AI works in two phases—the first phase, which involves “training” and the second phase which is “testing”. The model set uses the training data to set the parameters. The model uses the data from past examples, like data from patients or data with different examples, retrospectively. These parameters are then applied on the test sets. Various studies that have described the prognostic factors of OC are detected through AI by different biomarkers. Early diagnosis of the malignant lesion is good for patient survival rate and proper treatment therapy [12,13,14,15,16]. Many studies have been conducted using image analysis to smartphone-based OC detectors, based on AI algorithms. The AI technology facilitates the diagnosis, treatment, and management of patients with OC. AI reduces workload, complex data, and fatigue among physicians, for easy diagnosis [4,17]. The present systematic review intends to report on the application and role of AI-based technology in diagnosis and prediction of OC occurrence.

## 2. Materials and Methods

### 2.1. Search Strategy

In this systematic review, we followed the guidelines given by preferred reporting items for systematic reviews and meta-analyses extension, for the diagnostic test accuracy (PRISMA-DTA) [18]. Data search was mainly carried out through an electronic search in several renowned databases, which mainly included PubMed, Google Scholar, Scopus, Embase, Cochrane, Web of Science, and the Saudi Digital Library for articles that were published between January 2000 to March 2021. Index words like “artificial intelligence; oral cancer diagnosis; oral cancer prediction; oral cancer prognosis; deep learning; and machine learning” were used for searching the articles. Boolean operators (AND, OR) with language filters for English were used for searching articles in most electronic databases.

Simultaneously, a manual search for the research articles was also conducted along with the electronic search. A search for articles was carried out for the relevant citations from the reference list of previously retrieved articles in department and college libraries, where hard copies of the journals were available.

PICO (problem/patient, intervention/indicator, comparison, and outcome) elements were used for searching data on this topic (Table 1).

### 2.2. Study Selection

The electronic database search yielded 620 articles that were followed by hand searching, which yielded another 8 articles, which made a total of 628 articles. Initially, the articles chosen were based on relevance in the area of research, the title, and the abstract. Later, the articles were also manually checked for duplication by 2 members who were not involved in the preliminary search, which further eliminated 288 duplicated articles. Following this, 340 full-text articles were selected for data selection. The following eligibility criteria were applied at the next stage.

### 2.3. Inclusion and Exclusion Criteria

The articles were included according to the following inclusion criteria—(a) the article must be original research and must report on the AI technology; (b) quantifiable values that can be evaluated/analyzed should be mentioned in the article; and (c) the data used in evaluating these AI-based models should be mentioned. There was no limit set for the study design for inclusion in this systematic review.

The articles excluded were—(a) the articles in which AI innovation were not mentioned; (b) unpublished articles or conference papers that were uploaded online; (c) articles where full-text versions were not available; and (d) articles available in languages other than English.

### 2.4. Data Extraction

**Figure 1:**
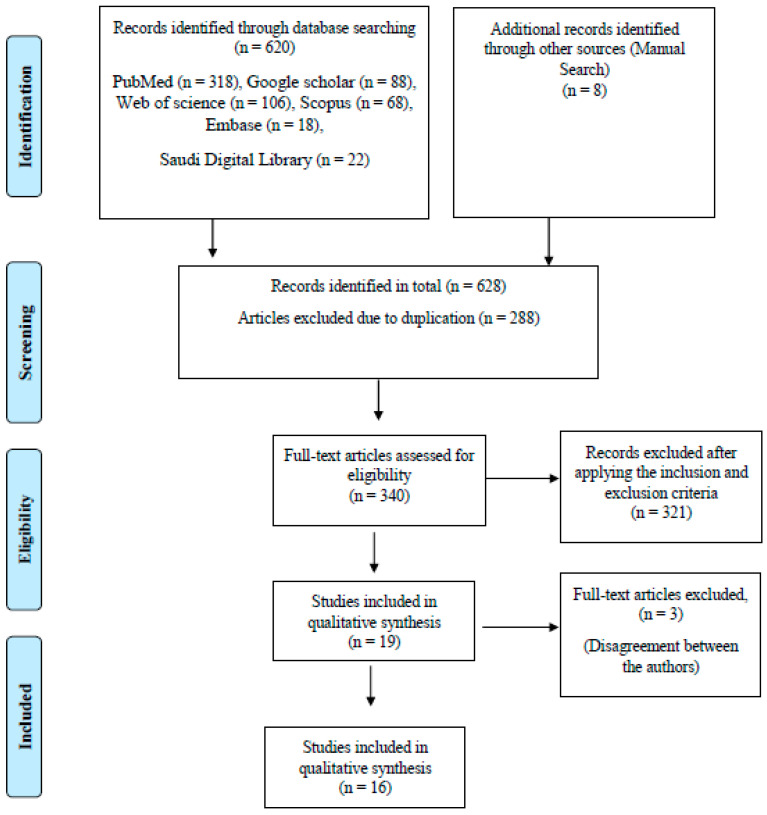
Flow chart for screening and selection of articles.

After applying the inclusion criteria, we filtered 12 articles out of the total. These 12 articles were considered to be potentially eligible articles for this systematic review, and were critically analyzed by the entire team. The details of the journal were covered before circulating them for critical analysis among authors. The QUADAS-2 tool was used for assessing the quality of the studies reporting on diagnostic accuracy. It has four domains which are assessed in terms of risk of bias and applicability concerns. The domains are patient selection, index test, reference standard, and flow and timing [18]. The authors disagreed with including 3 articles in this systematic review, as there was no mention of the reasonable data supporting the results and conclusions. Following this, the articles were further reduced to 16. The selection of the articles for qualitative synthesis for this systematic review is represented in the flow chart (Figure 1). The articles were further quantified with regards to the year of publication, to report on the trends in research that has been conducted on OC diagnosis and the prediction of prognosis, using the AI technology.

## 3. Results

Finally, 9 articles were critically analyzed for the extraction of the quantitative data. Most studies reported in the literature revealed that these studies were reported over the last 15 years. The trend showed a gradual increase in the studies reporting on the application of AI for OC diagnosis and the prediction of prognosis.

### 3.1. Qualitative Synthesis of the Included Studies

AI technology has been mainly applied for differentiating between normal, premalignant, and malignant conditions [19,20,21,22,23], predicting the likelihood of oral cancer incidence [24,25,26], prognosis, early detection of pre-cancerous and cancerous lesions [27,28,29,30], predicting the risk of recurrence [31,32], predicting the possibility of disease development from potential malignant lesion, and predicting the survival of patients [33,34].

In this systematic review, 4 studies were reported using convolutional neural networks (CNNs), and another 4 studies were reported using artificial neural networks (ANNs). These neural networks were mainly designed for assessing patient datasets, high-resolution cytology images, hyperspectral images, autofluorescence images (AFI), and white light imaging (WLI) (Table 2).

### 3.2. Risk of Bias Assessment and Applicability Concerns

**Figure 2:**
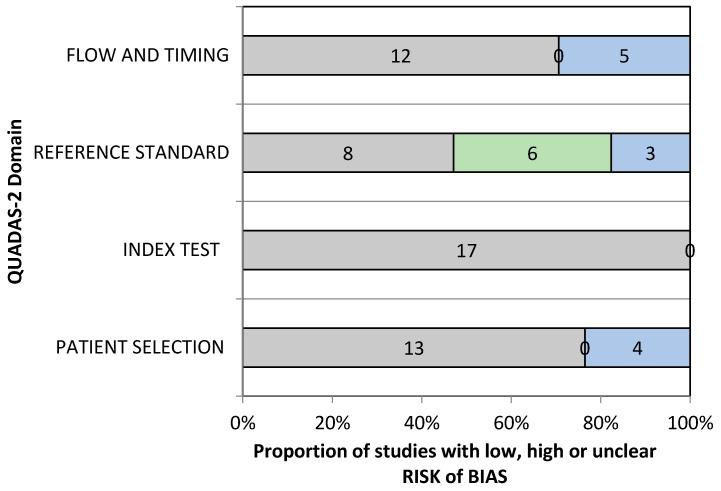
QUADAS-2 assessment of the individual risk of bias domains.

**Figure 3:**
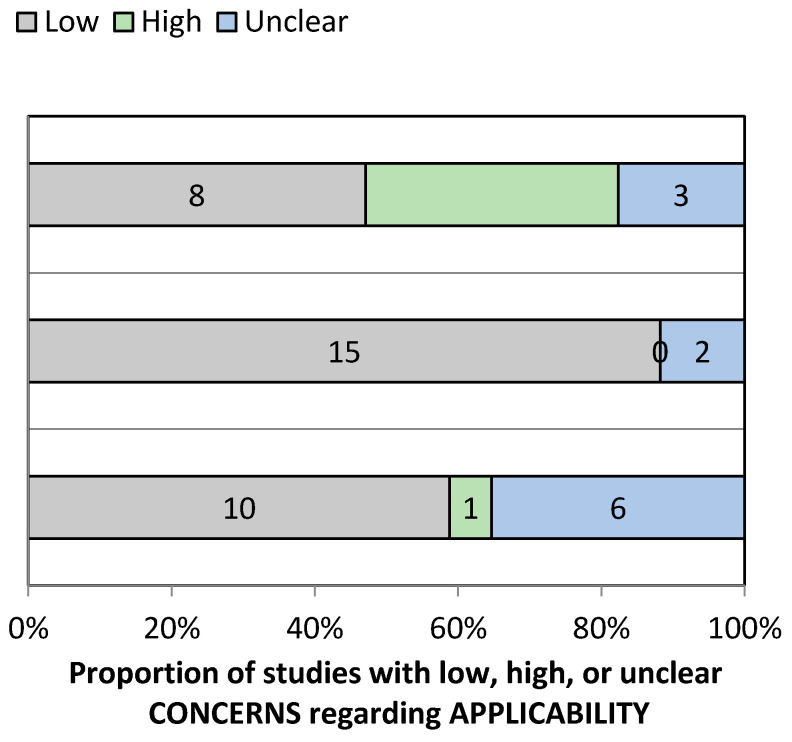
QUADAS-2 assessment of applicability concerns.

The QUADAS-2 assessment tool was used for assessing the quality and risk of bias of the included studies (Table S1). Most studies involved using photographic data as an input to the CNNs and ANNs, and hence, 76.47% of the included studies reported a low risk of bias for the patient-selection domain. However, in four studies, the patient-selection method was unclear. Since the data feeding in AI technology was highly standardized and there was no effect of flow and time frame on the final output, both the factors were categorized in a low-risk group. Nayak et al. used histopathology as the gold standard and studies by Tseng et al., Alabi et al., and Kim et al., were based on the prognostic outcome of the OSCC patients [19,27,33,34]. Hence, the reference standard in this situation was graded as low risk. Reference standard and the flow and timing domain were unclear in 17.64% and 29.41%, respectively. Hence, in this paper, a low risk of bias was reported in the index test (100%) and (70.58%) the inflow and timings. Under the risk of a bias arm of the QUADAS-2 tool, the applicability concern arms also showed 88.23% and 47.05% low risk of bias in the index test and the reference standard. However, patient selection and index test domain were unclear for 35.29% and 11.76% (Table S2, and Figure 2 and Figure 3).

## 4. Discussion

Oral cancer is one of the most prevalent cancer with high mortality, and it is a significant public health issue. Late diagnosis and high death rates are attributes of cancer around the world. According to the 2015 statistics of World Health Organization (WHO), cancer is the first or the second driving reason of death in almost 91 of 172 countries. The diagnosis and prediction of the reoccurrence of OC are the challenging factors, as AI involves complex data on etiology and risk factors [35,36,37].

AI is an exceptionally fresh development with a significant prognostic power, which allows clinicians to select appropriate treatment modalities. AI holds an incredible guarantee to empower clinicians to make noteworthy choices, depending on the immense amount of digitized data. Previous studies have applied machine-learning methods to huge patient datasets for early diagnosis and predicting the risk of occurrence of OC. 

AI has a more preferred advantage over existing techniques for detecting OC. It is a versatile innovation and can acquire additional information at any time. As AI calculations get information from new patients, they can merge this information into their dynamic datasets to improve their prescient exhibition and can reduce the burden of treatment and cost for patients [38]. There are two types of AI technologies, artificial neural networks (ANN) and convolution neural networks (CNN). The significant difference between the two is that in CNN, only the last layer of a neuron is completely associated. While in ANN, every neuron is associated with each different neuron [39]. This paper expects to examine the performance of these AI-based models that have reported on the diagnosis and prediction of the risk of occurrence of OC.

### 4.1. Artificial Intelligence in Detecting and Diagnosing Oral Cancer

As the late-stage disease has poor prognosis, early detection is important in OC patients. The data obtained from cytology images, fluorescent images, CT images, and depth of invasion can be used in AI learning tools, and OC can be diagnosed quickly with more accuracy. From our collected list of articles, 6 articles reported the application of AI-based computerized models for diagnosing OC. Several studies have carried out early detection of the advanced stage of OC and studies have reported that OC arise from different subsites of the oral cavity such as tongue, buccal mucosa, etc. This heterogeneity of oral malignant growth makes it difficult to be analyzed.

Sunny et al. conducted a study by ANN for early detection of OC, using tele cytology (TC), which is digitization of the cytology slides [29]. The efficacy of AI was compared with conventional cytology and histology; 11,981 prepossessed images were loaded for AI analysis, based on the risk stratification model. Results showed an accuracy of 80–84% in diagnosis, with no difference in tele cytology and conventional cytology detection, however, potentially malignant oral lesions were detected with low sensitivity, using tele cytology. The ANN-based model showed improved malignant detection accuracy to 93%, and a potentially malignant lesion to 73%. The study used the brush biopsy method for sample collection, which is less invasive, and this factor should also be considered while detecting cancer.

Jeyaraj et al. conducted a study in which OC was diagnosed based on a regression-based deep-learning algorithm for the characterization of oral malignant growth [30]. A deep-learning algorithm of CNN was developed in a computer-aided OC detecting system and 100 hyperspectral images (HIS) were analyzed. They observed a 91.4% sensitivity in detecting cancerous lesions using the regression-based algorithm, and the results were compared to the traditional algorithm using the same images. The quality of diagnosis was improved for the proposed model of the algorithm, as compared to the conventional.

Uthoff et al. conducted a study on detecting OC by using smartphone-based images and AI technology [28]. Based on the concept of point of care, smartphone-based images were developed. Autofluorescence and white light imaging were added to the pictures, and these pictures were stacked to AI algorithms for recognizing oral malignancy. A sum of 170 autofluoresced pictures was taken. This strategy was very convenient for application, and the accuracy was improved. However, the study needs to be conducted on a large population for further validation. A similar study was done by Nayak et al., using autofluorescent spectral images, and analysis was done using principal component analysis (PCA) and ANN [19]. PCA is computing based on principal components of data and the results from ANN performance was slightly better than the PCA. The advantage of this technique was that fluorescence spectroscopy image uses a minimally invasive technique and there is no need for biopsy [27,40]. In a study conducted by Musulin et al., AI showed better results in detecting OC, by using Histology images [21]. Similarly, in a study conducted by Kirubabai et al., CNN was better at differentiating malignant lesions as mild or severe, by using clinical images of patients [22].

Kann et al. applied deep-learning machines on 106 OC patients for the identification of nodal metastasis and tumor extra-nodal extension involvement [17]. The dataset comprised 2875 CT (computerized tomography) segmented lymph node samples. This study explored the capability of the deep-learning model to assist head and neck cancer patient management. For DNN, the area under the receiver operating characteristic curve (AUC) showed 0.91, which implied a higher accuracy. AUC represents the two-dimensional areas under the receiver operating characteristic curve (ROC). Similarly, Chang et al., reported an AUC of 0.90 for predicting the occurrence of OC, using AI based on genome markers [41]. In this study, logistic regression analysis was used to compare with AI. However, the study was conducted on 31 patients, which is a considerably less sample size, a study on a larger number of patients has to be carried out for better analysis.

### 4.2. Artificial Intelligence in Predicting the Occurrence of Oral Cancer

Currently, OC is treated with advanced treatment aids, however, the reoccurrence rate of OC is very high. Treatment of oral malignant growth relies on the stage of the disease. Lack of an evidence-on staging system may prompt deficient or pointless treatment. Different prognostic biomarkers and restorative targets have been proposed in ongoing periods, but they are not reproduced in the present cancer staging system. To date traditional statistical methods have been used for predicting OC, for example, cox proportional hazard (CPH), and it is not suitable for predicting conditions like OC.

Considering the complex ‘dataset’ of oral carcinoma, an AI-based anticipation prediction will give satisfied outcomes. Previous studies that used AI for predicting OC yielded excellent results [34,42,43].

Alabi et al. conducted a study on 311 patients in Brazil which compared four machine-learning algorithms in predicting the risk of reoccurrence of oral tongue squamous cell carcinoma [33]. These different machine-learning AI-based algorithms were based on support vector machine (SVM), naïve Bayes (NB), boosted decision tree (BDT), and decision forest (DF). All these algorithms showed improved accuracy in diagnosis, but the BDT algorithm showed the highest accuracy. However, the study included fewer samples, and more external algorithm data is required.

Shams et al. employed AI with the gene expression profile, to predict the occurrence of OC and also the transformation of oral potentially malignant lesions [31]. The study was conducted on 86 subjects, among them, 51 subjects developed OC and 31 subjects remained without malignancy. The study compared SVM, DNN, and multi-layer perception (MLP). Excellent results were obtained by deep-learning machines with 96.5% accuracy and 94% accuracy was obtained with MLP [43].

Chui et al., predicted the occurrence of cancer, based on clinical, pathological data, and compared linear regression (LR), BDT, SVM, and k-nearest neighbors (KNN) models, and concluded that BDT was the best model [26].

Tseng et al. determined the difference between symptoms exhibited by demised and survived OC patients [27]. The performance was compared between conventional logistic regression, decision tree, and ANN, and was conducted on 674 OC patients. Study used prognostic factors such as survival rate, death, cancer occurrence, and metastasis. The study concluded that the decision tree was easy to interpret and accuracy of the decision tree, and ANN was compared more to conventional logistic regression.

Rosma et al. tested the effectiveness of AI in predicting cancer based on the risk habits and demographic profiles in a Malaysian cohort [24]. Prediction of OC was compared between fuzzy regression model, fuzzy neural network prediction model, and clinician opinion. Fuzzy regression provides means when there is a lack of data and also provides a relationship between explanatory and response variables. The AI-based neural network and fuzzy regression model performed better in accuracy than human opinion, in predicting the OC.

## 5. Conclusions

AI is more accurate in diagnosing oral cancer as compared to the conventional method of diagnosis. Retrospective clinical data of patients may help in improving the AI-based diagnosis. Additionally, AI-based algorithms showed more accurate results in predicting the OC occurrence. More data and studies are needed to conduct AI-based algorithms to predict OC. The treatment of OC will not be effective if they are diagnosed at a later stage. Subsequently, early recognition techniques are required. The complex etiology and high recurrence rate make the investigation difficult. The patients can be classified as high- and low-risk groups, using accurate data from AI, which helps clinicians in planning and treatment, as compared to conventional methods. Patients can be directed with sensible advice and the clinicians can be guided with informed decisions.

## Figures and Tables

**Table 1:** Description of the PICO (P = Population, I = Intervention, C = Comparison, O = Outcome) elements.

Research question	What are the applications and performance of the artificial intelligence models that have been widely used in oral cancer diagnosis, and predicting the prognosis.
Population	Patients, clinical images, radiographs, datasets, and histological images.
Intervention	AI-based models for oral cancer diagnosis and predicting prognosis.
Comparison	Expert opinions and reference standards.
Outcome	Measurable or predictive outcomes such as accuracy, sensitivity, specificity, ROC = Receiver Operating Characteristic curve, AUC = Area Under the Curve, ICC = Intra-class Correlation Coefficient, PPV = Positive Predictive Values, and NPV = Negative Predictive Values.

**Table 2:** Details of the studies that have used AI-based models for oral cancer diagnosis and predicting the prognosis.

Sr. No.	Authors	Year of Publication	Algorithm Architecture	Study Design	Objective of the Study	No. of Images/Photographs for Testing	Study Factor	Modality	Comparison, If Any	Evaluation Accuracy/Average Accuracy	Results (+) Effective, (−) Non Effective (N) Neutral	Outcomes	Authors Suggestions/Conclusions
1	Nayak et al. [19]	2005	ANNs	Cross sectional study	Discriminating normal, potentially malignant, and malignant conditions using principal component analysis (PCA) and artificial neural network (ANN)	50	Differentiating normal, potentially malignant, and malignant	Recorded spectra	Principal component analysis (PCA)	Accuracy 98.3%, specificity of 100% and sensitivity 96.5%	(+) Effective	ANN is found to be slightly better than PCA	This model is efficient for real-time application.
2	Tseng et al. [27]	2015	ANNs	Cohort study	ANN for predicting oral cancer prognosis	-	Determining the differences between the symptoms shown in past cases	Datasets	Decision tree (DT)	Not Mentioned	(+) Effective	Both decision tree and artificial neural network models showed superiority to the traditional statistical model.	Decision tree models are relatively easier to interpret compared to artificial neural network models.
3	Uthoff et al. [28]	2017	CNN’s	Crosssectional study	AI-based deep (CNNs) for early detection of pre-cancerous and cancerous lesions	170	Detection of pre-cancerous and cancerous lesions	Autofluorescence imaging (AFI) and white light imaging (WLI)	Specialist’s diagnosis	Sensitivities 85%, specificities 88.75%, positive predictive values 87.67%, and negative predictive values 85.49	(+) Effective	CNN achieving high values of sensitivity, specificity, PPV, and NPV compared to the on-site specialist gold standard.	Performance should increase as additional images are collected.
4	Shams et al. [31]	2017	CNN’s	Cross sectional comparative study	Deep Neural Network (DNN) for predicting the possibility of oral cancer development in Oral potentially malignant lesion patients	10	Oral cancer development in Oral potentially malignant lesion patients	Datasets	Support Vector Machine (SVM), Regularized Least Squares (RLS), Multi-Layer Perception (MLP)	High accuracy 96%	(+) Effective	The results show high accuracy using DNN than SVM and MLP	None
5	Jeyaraj et al. [30]	2019	CNN’s	Cross sectional comparative study	Deep learning algorithm for an automated, computer-aided oral cancer-detecting system	100	Detection of pre-cancerous as benign and post cancerous as malignant region	Hyperspectral images	The traditional medical image classification algorithm	Accuracy of 91.4%, sensitivity 94% and a specificity of 91%	(+) Effective	The quality of diagnosis is increased by proposed regression-based partitioned CNN learning algorithm for a complex medical image of oral cancer diagnosis	This deep learning the algorithm can be easily deployed for providing an automatic medical image classifier without expert knowledge.
6	Fahed Jubair et al. [20]	2020	CNN’s	Crosssectional study	Develop a lightweight deep CNN using Efficient net-B0 transfer model CNN for binary classification of oral lesions into benign and malignant or potentially malignant using standard real-time clinical images	716	Detecting oral cancer	Clinical images	None	accuracy was 85.0% specificity, 84.5%, sensitivity 86.7%	(+) Effective	AI can improve the quality and reach of oral cancer screening and early detection.	This model of being small in size and need small computation power and memory capacity.
7	Sunny et al. [29]	2019	ANNs	Cross sectional comparative study	Artificial Neural Network (ANN) based risk-stratification model for early detection of oral potentially malignant (OPML)/malignant lesion.	82	Oral potentially malignant (OPML)/malignant lesion.	High-resolution cytology images	Conventional cytology and histology	84–86% Accuracy Sensitivity 93%	(+) Effective	ANN-based risk stratification model improved the detection sensitivity of malignant lesions (93%) and high-grade OPML (73%), increasing the overall accuracy by 30%.	This model can be an invaluable Point-of-Care (POC) tool for early detection/screening in oral cancer.
8	Jelena Musulin et al. [21]	2021	ANNs	Cross sectional comparative study	Diagnosing OC using the histological image of a biopsy	322	Detecting oral cancer	Histological image	ResNet50, ResNet101 Xception MobileNetv2	Xception and SWT resulted in the highest classification value of 0.96 (σ = 0.042) AUCmacro	(+) Effective	The AI-based system has great potential in the diagnosis of OSCC	This cell shape and size, pathological mitoses, tumor-stroma ratio and the distinction between early and advanced-stage OSCCs
9	M. Praveena Kirubabai et al. [22]	2021	CNN	Cross sectional study	To classify the oral images into either normal or abnormal images and diagnosed into ‘Mild’ or ‘Severe’ using a deep learning algorithm	160	Detecting oral cancer	Oral images	None	accuracy was 99.7%, 98.6% of sensitivity, 99.1% of specificity, and 99.7%	(+) Effective	CNN has high accuracy in detecting OC	None
10	Jyoti Rathod et al. [23]	2019	CNN’s	Cross sectional comparative study	Classify different stages of oral cancer using machine learning techniques	-	Diagnosing and classifying the premalignant lesion	Data set	SVM, KNN, MLP RSF, and Logistic Regression	DT 90.68%, RSF 91%, SVM 88%, KNN 85%, MLP 81% and Logistic Regression gives 80% of accuracy	(+) Effective	DT and RSF produced the same accuracy results	classification of oral cancer can be classified efficiently with help of Random Forest and Decision Tree
11	Alabi et al. [33]	2019	ANNs	Cross sectional comparative study	Comparing the performance of four machine learning Models (ML) for Predicting Risk of recurrence of oral tongue squamous cell carcinoma (OTSCC)	311	Prediction of reoccurrence	Patient datasets	5 Prognostic significance of the depth of invasion (DOI).	Accuracy of 68% for Support Vector Machine (SVM), 70% Naive Bayes (NB), 81% Boosted Decision Tree (BDT) and 78% Decision Forest (DF)	(+) Effective	Best classification accuracy was achieved with the boosted decision tree algorithm. These models outperformed the DOI-based approach	Machine algorithms should be considered in medical applications.
12	Kim et al. [35]	2019	CNNs	Retrospective study	Deep learning-based survival prediction method in oral squamous cell carcinoma (SCC) patients	255	Survival prediction	Datasets	Random Survival Forest (RSF) and the Cox proportional hazard model (CPH)	c-index of testing sets reaching 0.781	(+) Effective	This AI model displayed the best performance among the three models	This model can be effective in predicting with higher accuracy and can guide clinicians both in choosing treatment options and avoiding unnecessary treatments
13	Anwar Alhazmi et al. [25]	2020	ANNs	Crosssectional study	To develop (ANN) based model in predicting OC	73	Predicting risk of developing OC	Datasets	None	Accuracy of 78.95%	(+) Effective	ANN could perform well in estimating the probability of malignancy	More cohort studies are required based on this model
14	Chui S. Chu et al. [26]	2021	CNN’s	Cross sectional comparative study	To evaluate the ability of supervised machine learning models to predict disease outcome	467	Predicting risk of developing OC	Clinicopathological data	linear regression (LR), DT, SVM, and k-nearest neighbors (KNN) models	70.59% accuracy (AUC 0.67), 41.98% sensitivity, and a high specificity of 84.12%.	(+) Effective	CNN’s DT model was most successful in identifying “true positive” progressive disease	AI models in this study have shown promise in predicting progressive OSCC disease outcomes
15	Rosma et al. [24]	2010	ANNs	Cross sectional comparative study	Performances of the two artificial Intelligent prediction models when compared with a group of oral cancer clinicians.	171	Predicting the likelihood of an individual developing oral cancer	Datasets	27 oral cancer clinicians	Mean accuracy, sensitivity, and specificity of the models were 59.9, 45.5, and 85.3 for fuzzy neural network models; 63.1, 54.2, and 78.6 for oral cancer clinicians predictions and 67.5, 69.0 and 64.7 for fuzzy regression prediction models.	(+) Effective	Fuzzy regression and fuzzy neural network performed better than oral cancer clinicians	These neural network models provide a suitable alternative to human expert prediction in predicting oral cancer susceptibility.
16	Omar A. Karadaghy et al. [32]	2019	CNN’s	Crosssectional study	To develop a prediction DT model using machine learning for 5-year overall survival among patients with OSCC	33, 065	Predicting OSCC	Dataset	None	accuracy was 71%, precision was 71%,	(+) Effective	AI better in predicting OSCC	AI learning may play in individual patient risk estimation in the the era of big data.

ANNs: Artificial Neural Networks, CNNs: Convolutional Neural Networks, DNNs: Deep Neural Networks, and c-index: concordance index.

## Data Availability

Data is contained within the article.

## Supplementary Materials

The following are available online at https://www.mdpi.com/article/10.3390/diagnostics11061004/s1, Table S1: Quality assessment (QUADAS 2) summary of the risk of bias; Table S2: Quality assessment (QUADAS 2) summary of applicability concerns.
