# Supplementary material for: Application and Performance of Artificial Intelligence Technology in Oral Cancer Diagnosis and Prediction of Prognosis: A Systematic Review
Source: Diagnostics (Basel). 2021 May 31;11(6):1004. doi: 10.3390/diagnostics11061004 (PMC8227647; doi:10.3390/diagnostics11061004)
Supplement: Supplementary file 1 [file diagnostics-11-01004-s001.zip › diagnostics-1227094-supplementary.pdf]

Table S1. Quality assessment (QUADAS 2) summary of the risk of bias.

	PATIENT SELECTION	INDEX TEST	REFERENCE STANDARD	FLOW AND TIMING
Nayak et al [1]	Low	Low	Low	Low
Rosma et al [2]	Low	Low	High	Low
Tseng et al [3]	Low	Low	Low	Low
Uthoff et al [4]	Low	Low	High	Low
Shams et al [5]	Unclear	Low	Unclear	Unclear
Alabi et al [6]	Low	Low	Low	Low
Sunny et al [7]	Low	Low	High	Unclear
Jeyaraj et al [8]	Low	Low	Unclear	Low
Kim et al [9]	Low	Low	Low	Low
Alhazmi et al [10]	Low	Low	Low	Low
Chu et al. [11]	Low	Low	Low	Low
Jubair et al. [12]	Low	Low	Low	Low
Musulin et al [13]	Low	Low	High	Low
Rathod et al. [14]	Unclear	Low	Unclear	Unclear
Lavanya et al. [15]	Unclear	Low	High	Unclear
Kirubabai et al. [16]	Unclear	Low	High	Unclear
Karadaghy et al. [17]	Low	Low	Low	Low

Table S2. Quality assessment (QUADAS 2) summary of applicability concerns.

	PATIENT SELECTION	INDEX TEST	REFERENCE STANDARD
Nayak et al [1]	High	Low	Low
Rosma et al [2]	Unclear	Unclear	High
Tseng et al [3]	Low	Low	Low
Uthoff et al [4]	Low	Low	High
Shams et al [5]	Unclear	Low	Unclear
Alabi et al [6]	Low	Low	Low
Sunny et al [7]	Unclear	Unclear	High
Jeyaraj et al [8]	Low	Low	Unclear
Kim et al [9]	Low	Low	Low
Alhazmi et al [10]	Low	Low	Low
Chu et al. [11]	Low	Low	Low
Jubair et al. [12]	Low	Low	Low
Musulin et al [13]	Low	Low	High
Rathod et al. [14]	Unclear	Low	Unclear
Lavanya et al. [15]	Unclear	Low	High
Kirubabai et al. [16]	Unclear	Low	High
Karadaghy et al. [17]	Low	Low	Low

References

1. Nayak, G.S.; Kamath, S.; Pai, K.M.; Sarkar, A.; Ray, S.; Kurien, J.; D'Almeida, L.; Krishnanand, B.R.; Santhosh, C.; Kartha, V.B.; et al. Principal component analysis and artificial neural network analysis of oral tissue fluorescence spectra: Classification of normal premalignant and malignant pathological conditions. *Biopolymers* **2006**, *82*, 152–166, doi:10.1002/bip.20473.
2. Rosma, M.D.; Sameem, A.K.; Basir, A.; Siti Mazlipah, I.; Norzaidi, M.D. The use of artificial intelligence to identify people at risk of oral cancer: Empirical evidence in Malaysian university. *Int. J. Sci. Res. Educ.* **2010**, *3*, 10–20.
3. Tseng, W.T.; Chiang, W.F.; Liu, S.Y.; Roan, J.; Lin, C.N. The Application of Data Mining Techniques to Oral Cancer Prognosis. *J. Med. Syst.* **2015**, *39*, 59–66, doi:10.1007/s10916-015-0241-3.
4. Uthoff, R.D.; Song, B.; Sunny, S.; Patrick, S.; Suresh, A.; Kolur, T.; Keerthi, G.; Spires, O.; Anbarani, A.; Wilder-Smith, P.; et al. Point-of-care, smartphone-based, dual-modality, dual-view, oral cancer screening device with neural network classification for low-resource communities. *PLoS ONE* **2018**, *13*, 1–21, doi:10.1371/journal.pone.0207493.
5. Shams, W.K.; Htike, Z.Z. Oral Cancer Prediction Using Gene Expression Profiling and Machine Learning. *Int. J. Appl. Eng. Res.* **2017**, *12*, 4893–4898.
6. Alabi, R.O.; Elmusrati, M.; Sawazaki-Calone, I.; Kowalski, L.P.; Haglund, C.; Coletta, R.D.; Mäkitie, A.A.; Salo, T.; Almangush, A.; Leivo, I. Comparison of supervised machine learning classification techniques in prediction of locoregional recurrences in early oral tongue cancer. Int. J. Med. Inform. 2020, 136, 104068, doi:10.1016/j.ijmedinf.2019.104068.
7. Sunny, S.; Baby, A.; James, B.L.; Balaji, D.; N. V., A.; Rana, M.H.; Gurpur, P.; Skandarajah, A.; D'Ambrosio, M.; Ramanjinappa, R.D.; et al. A smart tele-cytology point-of-care platform for oral cancer screening. PLoS ONE 2019, 14, 1–16, doi:10.1371/journal.pone.0224885.
8. Jeyaraj, P.R.; Samuel Nadar, E.R. Computer-assisted medical image classification for early diagnosis of oral cancer employing
9. Kim, D.W.; Lee, S.; Kwon, S.; Nam, W.; Cha, I.H.; Kim, H.J. Deep learning-based survival prediction of oral cancer patients. Sci. Rep. 2019, 9, 1–10, doi:10.1038/s41598-019-43372-7.
10. Alhazmi, A.; Alhazmi, Y.; Makrami, A.; Masmali, A.; Salawi, N.; Masmali, K.; Patil, S. Application of artificial intelligence and machine learning for prediction of oral cancer risk. J. Oral Pathol. Med. 2021, 1–7, doi:10.1111/jop.13157.
11. Chu, C.S.; Lee, N.P.; Adeoye, J.; Thomson, P.; Choi, S. Machine learning and treatment outcome prediction for oral cancer. J. Oral Pathol. Med. 2020, 49, 977–985, doi:10.1111/jop.13089.
12. Jubair, F.; Al-karadsheh, O.; Malamos, D.; Al Mahdi, S.; Saad, Y.; Hassona, Y. A novel lightweight deep convolutional neural network for early detection of oral cancer. Oral Dis. 2021, 00, 1–8, doi:10.1111/odi.13825.
13. Musulin, J.; Štifanić, D.; Zulijani, A.; Ćabov, T.; Dekanić, A.; Car, Z. An enhanced histopathology analysis: An ai-based system for multiclass grading of oral squamous cell carcinoma and segmenting of epithelial and stromal tissue. Cancers 2021, 13, 1784, doi:10.3390/cancers13081784.
14. Rathod, J.; Sherkay, S.; Bondre, H.; Sonewane, R.; Deshmukh, D. Oral Cancer Detection and Level Classification Through Machine Learning. Int. J. Adv. Res. Comput. Commun. Eng. 2020, 9, 177–182, doi:10.17148/IJARCCE.2020.9335.
15. Lavanya L.; Chandra, J. Oral Cancer Analysis Using Machine Learning Techniques. Int. J. Eng. Res. Technol. 2019, 12, 596–601.
16. Kirubabai, M.P.; Arumugam, G. View of Deep Learning Classification Method to Detect and Diagnose the Cancer Regions in Oral MRI Images. Med. Legal Update 2021, 21, 462–468.
17. Karadaghy, O.A.; Shew, M.; New, J.; Bur, A.M. Development and Assessment of a Machine Learning Model to Help Predict Survival among Patients with Oral Squamous Cell Carcinoma. JAMA Otolaryngol. Head Neck Surg. 2019, 145, 1115–1120, doi:10.1001/jamaoto.2019.0981.
